# Immunotherapeutic treatment of inflammation in mice exposed to methamphetamine

**DOI:** 10.3389/fpsyt.2023.1259041

**Published:** 2023-11-09

**Authors:** Jennifer M. Loftis, Sankrith Ramani, Evan J. Firsick, Rebekah Hudson, Anh Le-Cook, Kevin S. Murnane, Arthur Vandenbark, Renee L. Shirley

**Affiliations:** ^1^Research and Development Service, Veterans Affairs Portland Health Care System, Portland, OR, United States; ^2^Department of Psychiatry, Oregon Health & Science University, Portland, OR, United States; ^3^Department of Behavioral Neuroscience, Oregon Health & Science University, Portland, OR, United States; ^4^Methamphetamine Research Center, Oregon Health & Science University, Portland, OR, United States; ^5^Department of Pharmacology, Toxicology and Neuroscience, Louisiana State University Health Sciences Center at Shreveport, Shreveport, LA, United States; ^6^Louisiana Addiction Research Center, Louisiana State University Health Sciences Center at Shreveport, Shreveport, LA, United States; ^7^Department of Psychiatry and Behavioral Medicine, Louisiana State University Health Sciences Center at Shreveport, Shreveport, LA, United States; ^8^Department of Neurology, Oregon Health & Science University, Portland, OR, United States; ^9^Department of Molecular Microbiology and Immunology, Oregon Health & Science University, Portland, OR, United States; ^10^Virogenomics BioDevelopment, Inc., Portland, OR, United States

**Keywords:** addiction, chemokine, drug discovery, frontal cortex, inflammation, methamphetamine, MIP-2, psychostimulant

## Abstract

**Introduction:**

Currently, there are no FDA-approved medications to treat methamphetamine addiction, including the inflammatory, neurotoxic, and adverse neuropsychiatric effects. We have shown that partial (p)MHC class II constructs (i.e., Recombinant T-cell receptor Ligand – RTL1000), comprised of the extracellular α1 and β1 domains of MHC class II molecules linked covalently to myelin oligodendrocyte glycoprotein (MOG)-35-55 peptide, can address the neuroimmune effects of methamphetamine addiction through its ability to bind to and down-regulate CD74 expression, block macrophage migration inhibitory factor (MIF) signaling, and reduce levels of pro-inflammatory chemokine ligand 2 (CCL2). The present study evaluated the effects of our third-generation pMHC II construct, DRmQ, on cognitive function and concentration of inflammatory cytokines in the frontal cortex, a region critical for cognitive functions such as memory, impulse control, and problem solving.

**Methods:**

Female and male C57BL/6J mice were exposed to methamphetamine (or saline) via subcutaneous (s.c.) injections administered four times per day every other day for 14 days. Following methamphetamine exposure, mice received immunotherapy (DRmQ or ibudilast) or vehicle s.c. injections daily for five days. Cognitive function was assessed using the novel object recognition test (NORT). To evaluate the effects of immunotherapy on inflammation in the frontal cortex, multiplex immunoassays were conducted. ANOVA was used to compare exploration times on the NORT and immune factor concentrations.

**Results:**

*Post hoc* analysis revealed increased novel object exploration time in MA-DRmQ treated mice, as compared to MA-VEH treated mice (non-significant trend). One-way ANOVA detected a significant difference across the groups in the concentration of macrophage inflammatory protein-2 (MIP-2) (*p* = 0.03). *Post hoc* tests indicated that mice treated with methamphetamine and DRmQ or ibudilast had significantly lower levels of MIP-2 in frontal cortex, as compared to mice treated with methamphetamine and vehicle (*p* > 0.05).

**Discussion:**

By specifically targeting CD74, our DRQ constructs can block the signaling of MIF, inhibiting the downstream signaling and pro-inflammatory effects that contribute to and perpetuate methamphetamine addiction.

## 1. Introduction

Methamphetamine is an amphetamine-based stimulant drug that can result in psychosis (1), depression (2, 3), anxiety (4, 5), and cognitive deficits (6, 7). Its misuse results in chronic, relapsing diseases that have given methamphetamine use disorders increasing global prevalence. In fact, an estimated 27 million people globally used methamphetamine in 2019 (3). Afflicted individuals often experience a range of neuropsychiatric deficits in episodic memory, executive function, and emotional regulation (7, 8). Consequently, increased relapse rates, lower treatment retention rates, and reduced daily functioning oftentimes result from the mental and behavioral health problems associated with methamphetamine misuse. New interventions are needed to help people regain the functions that methamphetamine altered and refrain from continued use of the drug. A growing body of research illustrates the critical role that neuroimmune pathways play in the propagation of neuronal injury that leads to the neuropsychiatric symptoms characteristic of methamphetamine use disorders (9–11). Approaches implementing immunotherapeutic, anti-inflammatory strategies in preclinical (12–18) and clinical trials have shown encouraging results in addressing addictive behavior and associated neuropsychiatric impairments (19–21).

In this study, we tested two immunotherapeutic strategies for the treatment of methamphetamine-induced cognitive impairments and inflammation. The first was the mouse-based partial major histocompatibility complex (pMHC) class II construct DRmQ (DRα1(L50Q)-mMOG-35-55) and the second was the phosphodiesterase inhibitor ibudilast (3-isobutyryl-2-isopropylpyrazolo-[1,5-a]pyridine). DRmQ and similar pMHCs have been shown to have neuroprotective and anti-inflammatory effects in several conditions, such as experimental stroke (22) and multiple sclerosis (23). DRmQs bind to and downregulate expression of CD74, a receptor that triggers a signaling cascade leading to neuroinflammation. CD74 is additionally the primary target receptor for macrophage migration inhibitory factor (MIF), a pro-inflammatory cytokine that is responsible for mediating the inflammatory effects in alcohol use disorders (24), depression (25), and neurodegenerative diseases (26). Ibudilast is another anti-inflammatory compound that inhibits MIF (27) and has been tested, with mixed results, for the treatment of methamphetamine use disorder (28, 29). Ibudilast is a non-selective phosphodiesterase inhibitor that also suppresses the production of nitric oxide, reactive oxygen species, interleukin (IL)-1β, IL-6, and tumor necrosis factor-alpha (TNF-α) and enhances the production of anti-inflammatory factors, including nerve growth factor, glia-derived neurotrophic factor, and neurotrophin-4 in activated microglia (30, 31). These mechanisms may be therapeutic, given that methamphetamine can cause persistent microglia activation in humans recently abstinent from methamphetamine use (32).

The purpose of this study was to test the effects of DRmQ and ibudilast in improving cognitive function and reducing inflammation in mice exposed to a neurotoxic methamphetamine regime (described in section 2.2.1 Methamphetamine exposure). To accomplish this goal, mice were exposed to methamphetamine or saline, followed by treatment with either DRmQ, ibudilast, or vehicle and behavioral testing (novel object recognition test). Luminex multiplex assays were used to measure concentrations of pro-and anti-inflammatory cytokines in the frontal cortex, a region critical for addiction-relevant cognitive functions such as memory, impulse control, and problem solving (33–36). Based on past studies testing MHC class II recombinant T-cell receptor ligands (RTLs) (14, 15) and ibudilast (13, 37), we hypothesized that these immunotherapeutic treatments would improve cognitive function and decrease concentrations of inflammatory cytokines, therefore combatting the neurotoxic effects of methamphetamine exposure.

## 2. Materials and methods

### 2.1. Animals

Ninety-six female and male C57BL/6 J mice [Jackson Laboratories (Bar Harbor, ME, United States); average age of 3 months and body weights (SD) of 21.5 (3.7) grams (g)] were tested in two cohorts that investigated the effects of immunotherapies (DRmQ or ibudilast) on cognitive function (cohort 1) and inflammation (cohort 2). For cohort 1, 48 male mice were assigned to one of eight treatment groups: (1) MA-VEH1: mice with methamphetamine (MA) exposure and without immunotherapy (received vehicle for DRmQ) (*n* = 3), (2) MA-VEH2: mice with methamphetamine exposure and without immunotherapy (received vehicle for ibudilast) (*n* = 3), (3) MA-DRmQ: mice with methamphetamine exposure and DRmQ immunotherapy (*n* = 8), (4) MA-IBU: mice with methamphetamine exposure and with ibudilast immunotherapy (*n* = 7). Groups 5–8 were identical to groups 1–4 except that mice were administered saline (SAL) instead of methamphetamine: (5) SAL-VEH1 (*n* = 4), (6) SAL-VEH2 (*n* = 4), (7) SAL-DRmQ (*n* = 8), and (8) SAL-IBU (*n* = 8). For cohort 2, 48 female mice were assigned to one of eight treatment groups, identical to that of cohort 1: (1) MA-VEH1 (*n* = 4), (2) MA-VEH2 (*n* = 4), (3) MA-DRmQ (*n* = 8), (4) MA-IBU (*n* = 8), (5) SAL-VEH1 (*n* = 4), (6) SAL-VEH2 (*n* = 4), (7) SAL-DRmQ (*n* = 8), and (8) SAL-IBU (*n* = 8). For cohort 1, there was attrition within the MA-IBU, MA-VEH1 and MA-VEH2 groups due to morbidity and mortality (*n* = 1 for each group) (Table 1).

**Table 1:** Cohort numbers and average weights of mice in the experimental groups before and after methamphetamine exposure and immunotherapy.

Treatment group	*n*	Baseline	Post-MA exposure	Post-immunotherapy
**Cohort 1 (*n* = 45)**				
SAL-VEH	8	25.0 (1.3)	26.2 (1.4)	27.1 (1.5)
SAL-DRmQ	8	25.0 (1.2)	26.5 (0.9)	26.9 (0.7)
SAL-IBU	8	26.1 (2.2)	27.1 (2.1)	29.2 (1.9)
MA-VEH	6	24.9 (1.7)	25.7 (1.1)	27.0 (1.2)
MA-DRmQ	8	24.5 (1.3)	25.6 (1.8)	26.4 (1.5)
MA-IBU	7	25.2 (2.0)	26.1 (1.1)	27.4 (1.1)
**Cohort 2 (*n* = 48)**				
SAL-VEH	8	18.9 (1.0)	20.9 (1.3)	21.3 (1.3)
SAL-DRmQ	8	18.2 (0.6)	19.9 (0.6)	20.5 (0.5)
SAL-IBU	8	18.1 (1.0)	20.2 (1.6)	21.4 (1.1)
MA-VEH	8	18.5 (0.9)	20.4 (0.6)	21.3 (0.5)
MA-DRmQ	8	17.6 (1.0)	19.4 (0.6)	19.8 (0.4)
MA-IBU	8	18.0 (0.9)	20.1 (1.0)	21.6 (0.9)

Data shown are mean grams (SD). MA, methamphetamine; SAL, saline; IBU, ibudilast; SD, standard deviation; VEH, vehicle.

### 2.2. Experimental design

**Figure 1:**
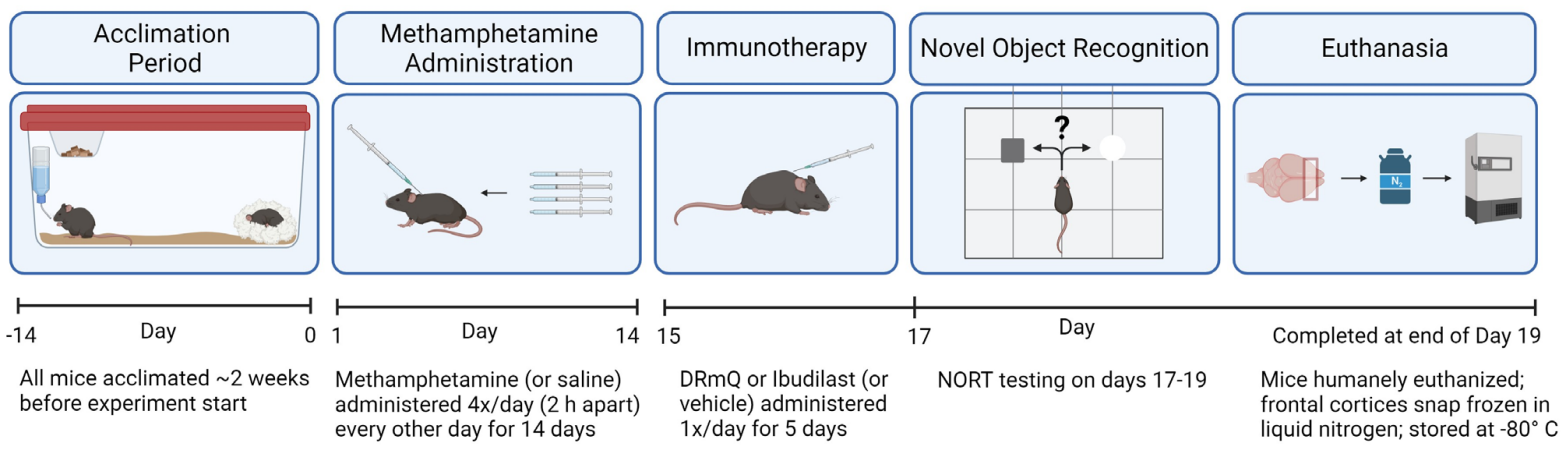
Timeline of the experimental design.

We evaluated the effects of methamphetamine and DRmQ or ibudilast immunotherapy on cognitive function and inflammation when treatment is administered during early remission from repeated binge exposure to methamphetamine (Figure 1). We have used this strategy previously (5, 15), as this parallels an ideal treatment approach in humans where treatment is provided during early recovery from addiction. This model mimics the CNS and neuropsychiatric impairments seen in humans with a history of methamphetamine addiction (3, 5, 33, 38) and provides a measurable signal to treat during an early remission period.

All experimental procedures were approved by the Veterans Affairs Portland Health Care System Institutional Animal Care and Use Committee. Experiments complied with the ARRIVE guidelines (39) and were carried out in accordance with the National Institutes of Health Guide for the Care and Use of Laboratory Animals.

#### 2.2.1. Methamphetamine exposure

Animals were exposed to methamphetamine every other day to model “binge and crash” use in humans [reviewed in (40)], which can result in long-term neural toxicity (41). This repeated methamphetamine exposure procedure using a 10 mg/kg dose recapitulates methamphetamine-induced neurotoxic effects observed in humans, including dopamine toxicity [e.g., (42)], and has been used previously without adverse consequences to the health of the animals (5, 15). Specifically, mice were exposed to methamphetamine via subcutaneous (s.c.) injections of methamphetamine (10 mg/kg) administered four times per day every other day for 14 days (7 days of methamphetamine exposure). On methamphetamine exposure days, each of the four injections was separated by 2 hours. The s.c. route was used for the methamphetamine injections to reduce potential trauma, as the needle penetrates only the skin. In addition, the rate of absorption for s.c. administration is slower than i.p. increasing the duration of methamphetamine exposure.

#### 2.2.2. Immunotherapy treatment

DRmQ (m = mouse) is comprised of the HLA-DRα1 domain with an L50Q (L = leucine; Q = glutamine) amino acid substitution (to enhance binding affinity for CD74) linked to an autoantigen peptide (myelin oligodendroglial cell glycoprotein, i.e., MOG-35-55 peptide). DRQ was derived by the Vandenbark lab from soluble major histocompatibility complex (MHC) Class II α1-β1-antigenic peptide constructs called Recombinant T-cell receptor Ligands (RTLs). The physical properties and mechanisms of action of RTLs have been described in detail previously [e.g., reviewed in (43)]. Ibudilast was obtained lyophilized (Selleck Chemicals, LLC., Houston, TX, United States) and reconstituted following vendor’s recommendations based on concentration needed for injection and pre-determined solubility information provided upon receipt of product.

Mice received daily s.c. injections of either DRmQ (100 μg), ibudilast (13 mg/kg), or vehicle [DRmQ vehicle: 20 mM Tris, pH 8.5; IBU vehicle: 35% polyethylene glycol (PEG) in saline] daily for 5 days. For cohort 2, the vehicle for ibudilast was modified (changed to 35% PEG in 10% DMSO in saline) to facilitate solubility.

#### 2.2.3. Novel object recognition test

Cognitive function was assessed using the novel object recognition test (NORT), with modifications based on published methods (44, 45). The NORT testing occurred across 3 days and consisted of habituation, training, and retention sessions. During the habituation session, mice were individually habituated to an open-field box (29 cm × 36 cm), without objects for 10 min. Following habituation, two of three objects (i.e., objects of similar size but different in shape) were symmetrically attached to the floor of the box and mice were given 10 min to explore (i.e., training session). Twenty-four hours later mice were placed back in the box for the retention session, and one of the objects was replaced with one not previously encountered. During the retention session, mice were given 5 min to explore. Behavior was recorded and measured using the EthoVision XT 10 video-tracking system (Noldus Information Technology Inc., Leesburg, VA, United States). Exploration of an object was defined as directing the nose at a distance ≤2 cm to the object and/or touching it with the nose, while turning around or sitting on the object was not considered exploration. Novel object exploration time was used to evaluate cognitive function (a measure of recognition memory). Exploratory preferences during the retention session were also calculated as (i) a preference index [amount of time spent exploring the novel object/the total time spent exploring both objects (during the retention session)] and (ii) a discrimination index [(amount of time spent exploring the novel object − familiar object)/(amount of time spent exploring the novel object + familiar object)], according to the method described (46, 47).

#### 2.2.4. Brain tissue preparation

Frontal cortices were microdissected on a cold block, immediately frozen in liquid nitrogen and then stored at −80°C until assayed. Brain tissue lysates were prepared in a 1:20 ratio, using 20 μL of lysis buffer [150 mM sodium chloride, 0.1% Triton X-100, 0.5% sodium deoxycholate, 0.1% SDS, 50 mM Tris-HCl, pH 8.0; Halt^™^ Protease and Phosphatase Inhibitor Single-Use Cocktail (100X) (Thermo Fisher Scientific, Waltham, MA, United States)] per 1 mg of tissue. Tissue from each animal was homogenized manually in a 1.5 mL Eppendorf tube on ice with a disposable pellet pestle. The samples were centrifuged at 12,000 rpm for 20 min at 4°C. Supernatants were aliquoted to fresh 1.5 mL Eppendorf tubes and used for multiplex immunoassays. Total protein concentration was determined using Pierce^™^ BCA (bicinchoninic acid) protein assay kits (Thermo Fisher Scientific, Waltham, MA, United States) and absorbance reader (BioRad 680).

#### 2.2.5. Multiplex immunoassay

MILLIPLEX MAP Mouse Cytokine/Chemokine Magnetic Bead Panel Immunology Multiplex Assay (MCYTOMAG-70K, Millipore Sigma, Burlington, MA, United States) was used to measure immune factors with a putative role in methamphetamine-induced CNS effects or CD74 signaling—i.e., monocyte chemoattractant protein-1 (MCP-1, a.k.a. CCL2) (38), interferon-gamma (IFN-γ) (48), IFN-γ induced protein-10 (IP-10) (49), interleukin-10 (IL-10) (50), IL-1β (51), IL-2 (15), macrophage inflammatory protein-1 alpha (MIP-1α) and MIP-2 (52). The immunoassay kit was performed according to the manufacturer’s instructions. Briefly, brain lysate samples were formulized to 2 μg/μL before being added into the 96-well plates (25 μL/well). Equal protein amounts were used in each well. The concentrations of cytokines were determined using a Luminex^®^ 200 system and 5-parameter curve-fitting method, as previously reported (15).

#### 2.2.6. Approach for unbiased data collection

Each mouse was assigned an ID number that did not denote group assignment. This information was uploaded in a secure database. Additionally, male and female research technicians worked with animals throughout experiments to prevent handling bias (53).

### 2.3. Statistical analyses

GraphPad Prism, version 8.1.2 software (La Jolla, CA, United States) was used for all statistical analyses. One-way analysis of variance (ANOVA) was used to compare body weight, cognitive function, and inflammatory factor concentration among the treatment groups, followed by Holm-Šidák’s multiple comparison or Kolmogorov–Smirnov *post hoc* tests, when appropriate. A *p*-value ≤0.05 denoted a statistically significant difference. Initial analyses found no significant differences between the VEH1 and VEH2 groups (as described in section 2.1), so VEH1 and VEH2 groups were combined for final analyses and reporting. Bar graphs shown in the figures illustrate group means ± SEM.

## 3. Results

### 3.1. Methamphetamine exposure, immunotherapy, and body weights

**Figure 2:**
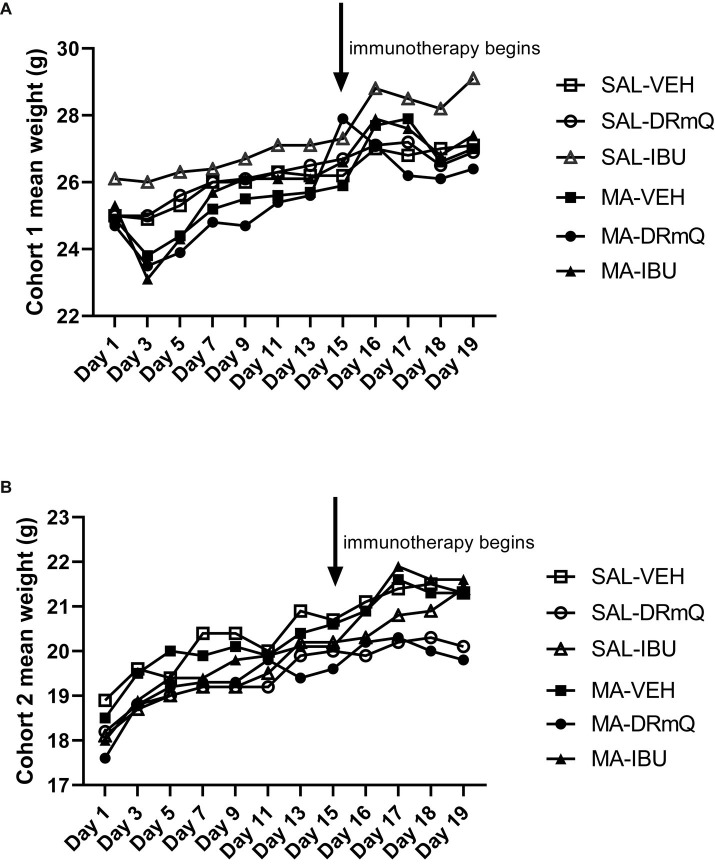
Body weights by treatment group over time during methamphetamine exposure and immunotherapy **(A)** cohort 1; **(B)** cohort 2.

The average body weights of mice in the experimental groups before and after methamphetamine exposure and immunotherapy are shown in Table 1. There were no significant differences in body weights among the eight groups for cohorts 1 and 2 at baseline, post-methamphetamine exposure, or post-immunotherapy. Figure 2 shows average body weights over time, illustrating that methamphetamine exposure resulted in an acute sign of stimulant activity for mice in cohort 1 (Figure 2A) but not cohort 2 (Figure 2B). Mice treated with methamphetamine lost an average of 6% of their body weight (1.5 g) during the first 3 days of drug exposure, as compared with saline-treated mice whose weights remained stable during the same time period.

### 3.2. Methamphetamine exposure, immunotherapy, and cognitive function

**Figure 3:**
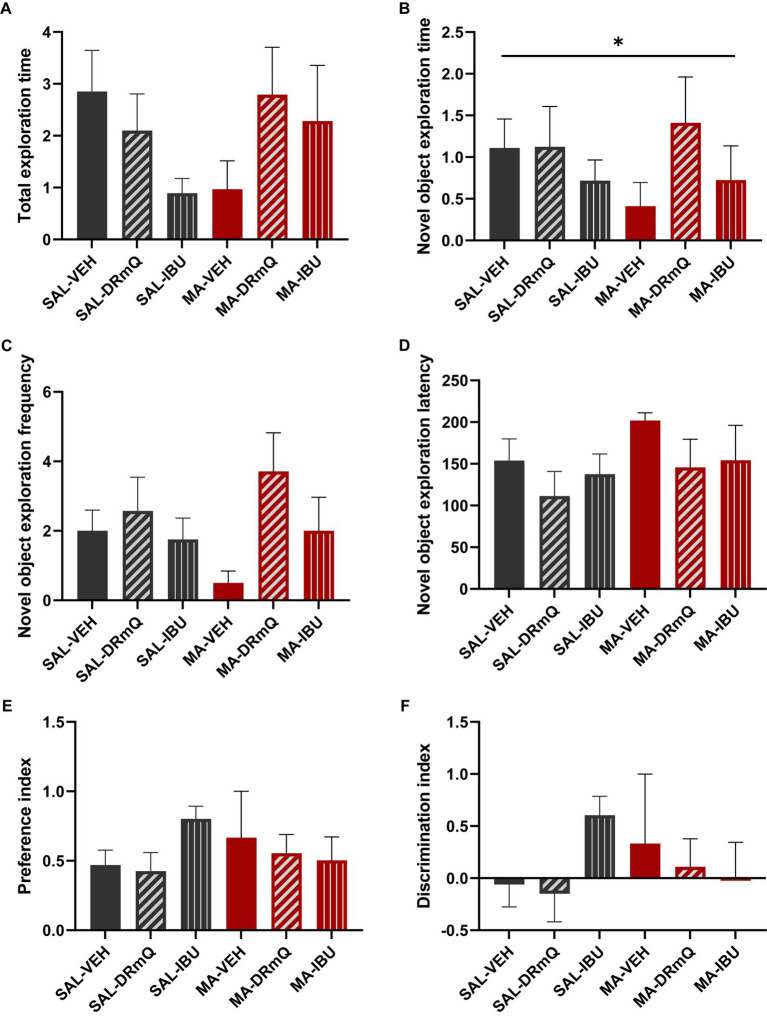
Cognitive impairment associated with methamphetamine exposure and immunotherapy treatment. Panels show results from the retention session of the novel object recognition test (NORT) conducted with mice in the six treatment groups. Data were analyzed using ANOVA, followed by *post hoc* comparisons as appropriate. **(A)** The total exploration time (i.e., time spent exploring both the familiar and novel objects during the retention trial) was not significantly different across the groups. **(B)** There was a significant between-group difference in the duration of time spent exploring the novel object (^*^p ≤ 0.05, one-way ANOVA); *post hoc* analyses indicated non-significant trending differences between SAL-VEH and MA-VEH (*p* = 0.06) and MA-VEH and MA-DRmQ (*p* = 0.07) groups. **(C,D)** The frequency of novel object recognition events (i.e., the number of times the mouse came within 2 cm of the novel object) and the latency to initially explore the novel object were not significantly different across groups. **(E,F)** The groups did not show significant differences in preference for the novel object or in abilities to discriminate the novel from the familiar object.

To evaluate the effects of methamphetamine exposure and immuotherapeutic treatments on cognitive function, mice were tested using the NORT, a behavioral test that assesses recognition memory function (44). Behavioral assessments were conducted over a three-day period (habituation, training, and retention sessions). During the retention session of the NORT, analyses indicated that across groups, mice spent similar amounts of time exploring both objects (total exploration: *F* = 1.40, *p* = 0.25) (Figure 3A). There was a significant between-group difference in the duration of time spent exploring the novel object (*p* = 0.05); *post hoc* analyses indicated non-significant trending differences between SAL-VEH and MA-VEH (*p* = 0.06) and MA-VEH and MA-DRmQ (*p* = 0.07) groups, with the MA-VEH group showing decreased exploration as compared to the SAL-VEH group and the MA-DRmQ group showing increased exploration as compared to the MA-VEH group (Figure 3B). The frequency of novel object recognition events (i.e., the number of times the mouse came within 2 cm of the novel object) was not significantly different across groups (*F* = 1.59, *p* = 0.19) (Figure 3C). There were also no statistically significant differences found for the time it took (latency) to initially explore the novel object (*F* = 0.45, *p* = 0.81) (Figure 3D). The groups did not show significant differences in preference for the novel object (*F* = 0.88, *p* = 0.51) (Figure 3E) or in abilities to discriminate the novel from the familiar object (*F* = 0.88, *p* = 0.51) (Figure 3F).

### 3.3. Methamphetamine exposure, immunotherapy, and frontal cortex inflammatory factors

**Figure 4:**
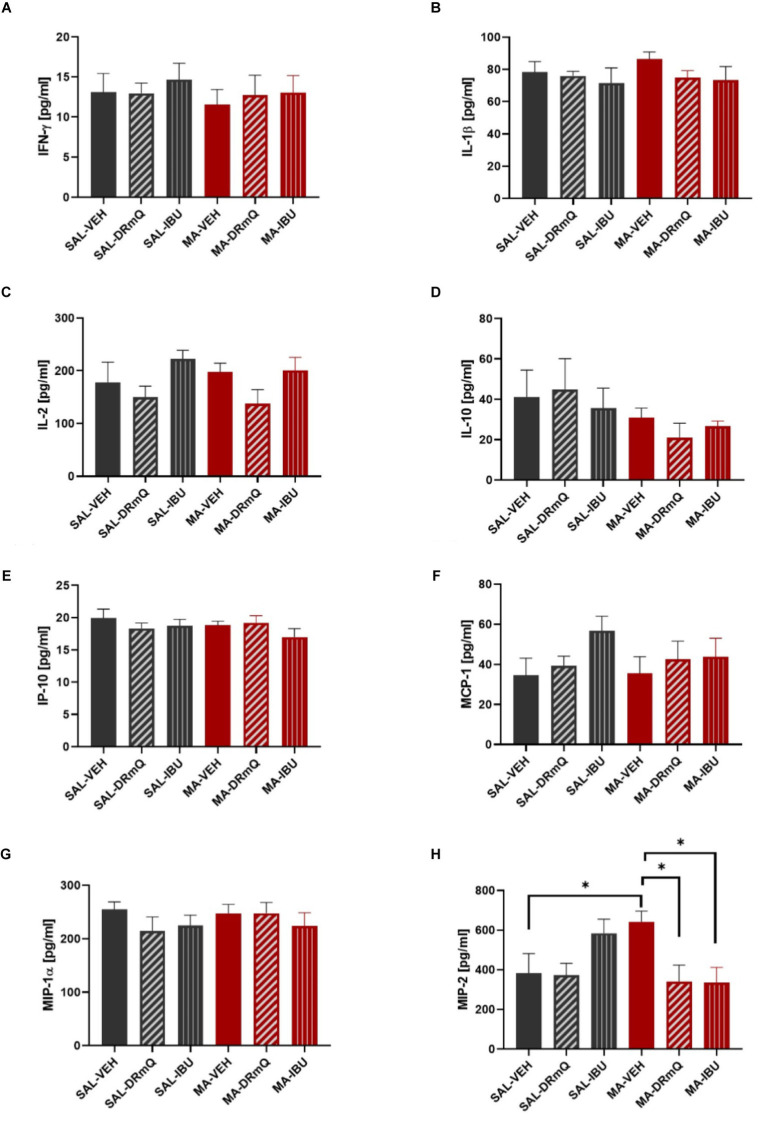
Cytokine levels in frontal cortices of mice treated with methamphetamine and immunotherapy. Panels **(A–H)** show mean (± SEM) concentrations of IFN-γ, IL-1β, IL-2, IL-10, IP-10, MCP-1, MIP-1α, and MIP-2, respectively. One-way ANOVA detected a significant difference across the groups (*p* = 0.03) for MIP-2; Holm-Šidák’s multiple comparisons test indicated that methamphetamine increased MIP-2 levels in the frontal cortex (MA-VEH group versus SAL-VEH group) and that methamphetamine-exposed animals treated with either DRmQ or IBU had significantly lower levels of MIP-2, as compared to methamphetamine-exposed mice treated with vehicle (^*^p < 0.05).

A panel of pro- and anti-inflammatory factors were measured in the frontal cortices of mice exposed to methamphetamine (or saline) and treated with immunotherapy (or vehicle). The mean concentrations of IFN-γ, IL-1β, IL-2, IL-10, IP-10, MCP-1, MIP-1α, and MIP-2 are shown in Figures 4A–H. One-way ANOVA detected a significant difference across the groups for MIP-2 (*F* = 2.879, *p* = 0.03). Holm-Šidák’s multiple comparisons test indicated that the MA-VEH group had significantly higher levels of MIP-2, as compared to the SAL-VEH group (*t* = 2.20, *p* = 0.05); methamphetamine-exposed animals treated with either DRmQ (*t* = 2.74, *p* < 0.05) or IBU (*t* = 2.78, *p* < 0.05) had significantly lower levels of MIP-2, as compared to vehicle (Figure 4H). There were no statistically significant between-group differences for the other immune factors measured (Figures 4A–G).

## 4. Discussion

A growing body of literature has shown that methamphetamine alters immune function in the peripheral and central nervous systems, increasing the expression of pro-inflammatory cytokines and chemokines that affect neuropsychiatric function. In this study, we tested two immunotherapeutic strategies for the treatment of methamphetamine-induced cognitive impairments and cortical inflammation. The frontal cortex is a particularly critical region for compulsive drug-taking behavior (54), craving, and decision-making in methamphetamine use disorder (55). Here, we show that novel object exploration time on the NORT, which measures declarative (episodic) memory [e.g., (56)] [a cognitive domain that is relevant to individuals with methamphetamine use disorder (57–59)], was reduced in MA-VEH treated mice, as compared to SAL-VEH treated mice (*p* = 0.06, non-significant trend). This effect of methamphetamine on cognitive function is consistent with other stimulant exposure. In mice, the pyrrolidine analog of methamphetamine, α-pyrrolidinopropiophenone (α-PPP), decreases exploration time in the NORT and impairs spontaneous alternation performance (a measure of spatial working memory) in the Y-maze (60). Similarly, in rats, chronic administration of methamphetamine has impairing effects on recognition memory (NORT) and spontaneous alternation performance (Y-maze) (61–63). With the goal of treating methamphetamine-induced cognitive impairments, we found that in contrast to ibudilast, DRmQ immunotherapy increased novel object exploration time, as compared to MA-VEH treated mice (*p* = 0.07, non-significant trend), suggesting the potential for improved cognitive function in our mouse model of methamphetamine binge exposure.

**Figure 5:**
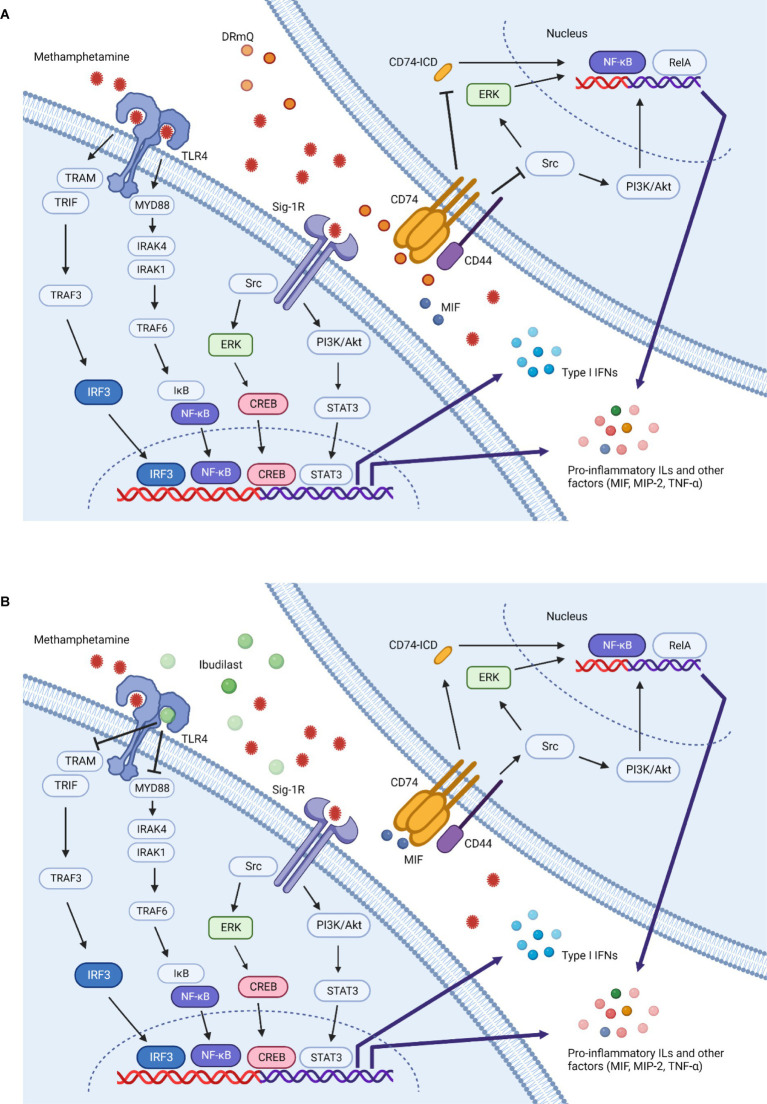
Methamphetamine-induced inflammation and immunotherapeutic mechanisms of action. Multiple mechanisms contribute to the inflammation induced by methamphetamine. This simplified schematic illustrates several potential inflammatory pathways and the effects of DRmQ and ibudilast on these signals. Methamphetamine binds to the sigma-1 receptor (Sig-1R) (67, 68) and Toll-like receptor 4 (TLR4) complex (69), triggering signaling pathways which ultimately upregulate the expression of inflammatory cytokines including interleukins (IL-1, IL-2, IL-6, IL-8), interferons (IFNs), MCP-1, MIF, MIP-2 [recently reviewed in (51)], and other factors relevant to methamphetamine-induced pathology, such as brain derived neurotrophic factor (BDNF) (70) and intracellular adhesion molecule-1 (ICAM-1) (71) (*not shown*). At the Sig-1R, methamphetamine activates downstream signaling pathways, including nuclear factor kappa light chain enhancer of activated B cells (NF-κB), mitogen-activated protein kinase (MAPK) and phosphoinositide 3-kinase (PI3K)/protein kinase B (Akt) pathways (72, 73). Rat primary astrocytes exposed to methamphetamine show increased expression of Sig-1R via the activation of Src, extracellular signal-regulated kinase 1/2 (ERK) (belongs to the MAPK family), and downstream cAMP response element binding protein (CREB) pathways (74). Activation of CREB promotes inflammation through expression of various inflammatory cytokines (75). Further, methamphetamine-induced microglial activation involves Sig-1R binding, reactive oxygen species (ROS) generation, activation of the MAPK and PI3K/Akt pathways, and pro-inflammatory protein expression (72, 76). At the TLR4 complex, methamphetamine can activate both myeloid differentiation primary response protein 88 (Myd88)-dependent and non Myd88-dependent pathways (77). In the Myd88-dependent pathway, signal transduction occurs through interleukin 1 receptor associated kinase 4 and 1 (IRAK4 and IRAK1) and TNF receptor associated factor 6 (TRAF6). The activation of TRAF6 leads to phosphorylation of inhibitors of nuclear factor κB kinases (IKKs), which in turn activates IκB. The activation of IκB leads to its degradation and the activation of NFκB, thereby mediating the production of pro-inflammatory cytokines (e.g., TNF-α, IL-1β and IL-6) (17, 78–80). In the non Myd88-dependent pathway, TLR4 triggers the activation of the TRIF-related adaptor molecule (TRAM), followed by the activation of TRAF3, TANK-binding kinase 1 (TBK1) and IKKε (*not shown*), which phosphorylates IFN regulatory factor 3 (IRF3). IRF3 then translocates to the nucleus and promotes the transcription of type 1 IFNs. In the later inflammatory response process, TRAM can also activate NF-κB (*not shown*) (72, 81, 82). **(A)** DRmQ is theorized to prevent methamphetamine-induced upregulation of pro-inflammatory factors by acting as a competitive inhibitor of CD74—the primary receptor for MIF. MIF binding to CD74 and recruits CD44 (cell surface adhesion receptor). Phosphorylation of CD74 and CD44 activates the Src-family tyrosine kinase Lck, in turn activating the ERK or PI3K/Akt signaling pathways and inducing the expression of pro-inflammatory cytokines through the NF-kB pathway. MIF also signals via the RelA (NF-κB-family pathway) pathway activating genes involved in inflammatory responses. The interaction of MIF and CD74 can also promote the cleavage of CD74 to produce CD74 intracellular domain (CD74-ICD), which is thought to provide a further activation signal to induce the production of several pro-inflammatory cytokines, including interleukins (IL-1, IL-2, IL-6, IL-8), TNF-α, and MCP-1 (83–85). Thus, DRmQ may disrupt the CD74 binding and subsequent pro-inflammatory signaling cascades. **(B)** Ibudilast can block methamphetamine-induced inflammation by acting as a competitive inhibitor of TLR4. TLR-4 blocking may lead to the reduced production of pro-inflammatory cytokines via pathways that also implicate NF-κΒ, IRAK1 and TRAF6. Additional anti-inflammatory mechanisms of ibudilast have been proposed (86) but for clarity are not shown in this figure.

The mouse-based pMHC construct DRmQ may be able to address the cognitive and neuroimmune effects of methamphetamine addiction via its anti-inflammatory effects and ability to promote remyelination. The pMHC moiety produces an antigen non-specific inhibitory effect after binding to and downregulating CD74 (the class II invariant chain) mainly on macrophages, including those that cross the BBB after CNS damage. Down regulation of CD74 expression blocks MIF signaling, promotes neuroprotection, inhibits recruitment of inflammatory cells to brain, and reduces inflammation (64–66) (Figure 5). In a recently published manuscript (67), the function of myelinated axons in two different white matter tracts (i.e., corpus callosum and optic nerves) were shown to improve in a mouse model of multiple sclerosis (EAE) following treatment with DRhQ. Myelin damage (92–95) is associated with methamphetamine exposure as research shows that there is altered expression of myelin sheath components [e.g., MOG, myelin basic protein (MBP)] following methamphetamine and cocaine exposure (96, 97) and other addictive substances are associated with the development of antibodies to MBP (98). Thus, this dual action of DRmQ is believed to contribute to its therapeutic effect and may explain why DRmQ showed a non-significant trend to improve cognitive function (*p*= 0.07), even though DRmQ and ibudilast both decreased MIP-2 levels (*p*< 0.05) (Figure 4H). The initial pMHC constructs (i.e. RTL1000) were designed to target MOG-35-55 specific T cells. The lack of the DR2 β1 domain in DRQ constructs enables the therapeutic to be administered to patients regardless of DR2 status but also likely reduces the ability of DRQ to function as a T cell receptor ligand.

Ibudilast has neuroprotective and immunomodulatory properties and has shown therapeutic benefit for substance use disorders, including methamphetamine use disorder. It is a relatively non-selective inhibitor of several phosphodiesterases (PDEs), including PDE3, PDE4, PDE10 and PDE11, as well as MIF and Toll-like receptor-4 (TLR-4) (31, 98, 99) (Figure 5). Ibudilast can reduce glial cell activation, pro-inflammatory cytokines levels (e.g., TNF-α, IL-1β and IL-6), and methamphetamine self-administration (13, 21, 31, 37). Ibudilast has also been shown to reduce methamphetamine-induced locomotor activity and stress-induced methamphetamine reinstatement (37). In clinical trials, ibudilast lessened craving for methamphetamine (29) but failed to demonstrate a significant difference between ibudilast and placebo in reducing methamphetamine use (28).

To assess inflammatory effects, we measured immune factors with evidence for a role in methamphetamine-induced CNS effects or CD74 signaling. Although some studies show increases in pro-inflammatory cytokines such as IFN-γ and IL-1β following methamphetamine exposure [reviewed in (51, 100)], we did not find significant methamphetamine-induced increases in these factors (Figures 4A,B). The timing of our sample collection and tissue type (frontal cortex) may have contributed to the different observations. However, we found that MIP-2, a factor which plays an important role in the progression and perpetuation of inflammation, was increased following methamphetamine (Figure 4H). Consistent with the anti-inflammatory properties of DRmQ and ibudilast, the concentration of MIP-2 in the frontal cortex was significantly lower in mice treated with methamphetamine and DRmQ or ibudilast, as compared to mice treated with methamphetamine and vehicle. MIP-2 is one of the CXC chemokines [also known as chemokine CXC ligand (CXCL2)] and is produced by a variety of cell types, such as glial cells, macrophages, monocytes, epithelial cells, and hepatocytes, in response to infection or injury (101, 102). Studies show associations between MIP-2 levels and organ inflammation and injury, such as in cortical damage (103), pneumonia (104), and alcohol-induced liver injury [reviewed in (101)]. MIP-2 is increased in rodent cortical cultures exposed to cocaine (105), but to date, there are no published reports on methamphetamine’s effects on MIP-2 expression. There are similarly limited data on the role of MIP-2 in cognitive function; however, one recent study found elevated concentrations of MIP-2 in peripheral blood and hippocampus in rats with cognitive dysfunction (106).

This present study contributes to research appreciating the importance of cognitive function in recovery from substance use disorders (107–110). Methamphetamine-induced cognitive deficits reflect changes in the underlying cortical, subcortical, and neuro-modulatory mechanisms that underpin cognition and can interfere with treatment outcomes (111, 112). Methamphetamine use has been linked to a broad range of cognitive deficits involving domains of complex attention, reasoning, memory, impulse control, and executive functions (57, 113). Of particular interest is the role that episodic memory and executive functions play in methamphetamine use. Findings suggest that individuals with methamphetamine dependence experience difficulty in the retrieval of future intentions, which has implications for recovery efforts and everyday functioning (114). Further, in a longitudinal study of participants with a history of methamphetamine use, those who relapsed to methamphetamine use showed worse episodic memory performance than those who remained abstinent as well as those with continued use, suggesting that relapse to methamphetamine use may affect episodic memory differently than it affects the other cognitive functions measured (59).

Our study had limitations. To assess cognitive function, we utilized only one behavioral test (i.e., NORT) and did not include other measures to assess different cognitive domains or general locomotor activity. Another limitation is that our neurochemical investigations measured immune factor levels but not neurotransmitter levels, particularly those affected by methamphetamine. It would be of interest to know, for example, how the immune factor changes observed relate to changes in monoamine neurochemistry or functional dynamics such as loss of tonic dopamine levels. Despite these limitations, the evidence reported herein contributes to a growing consensus that an immunotherapeutic approach has the potential to reduce the inflammatory and adverse neuropsychiatric effects of methamphetamine.

## Data availability statement

The raw data supporting the conclusions of this article will be made available by the authors, without undue reservation.

## Ethics statement

The animal study was approved by the Institutional Animal Care and Use Committee, VA Portland Health Care System, Portland, OR. The study was conducted in accordance with the local legislation and institutional requirements.

## Author contributions

JL: Conceptualization, Funding acquisition, Investigation, Resources, Supervision, Writing – original draft, Writing – review & editing. SR: Visualization, Writing – original draft. EF: Investigation, Methodology, Writing – review & editing. RH: Investigation, Writing – review & editing, Formal analysis. AL-C: Investigation, Writing – review & editing, Methodology. KM: Writing – review & editing. AV: Writing – review & editing, Resources. RS: Writing – review & editing, Conceptualization, Funding acquisition.
